# Maximum Likelihood-Based Iterated Divided Difference Filter for Nonlinear Systems from Discrete Noisy Measurements

**DOI:** 10.3390/s120708912

**Published:** 2012-06-27

**Authors:** Changyuan Wang, Jing Zhang, Jing Mu

**Affiliations:** 1 School of Computer Science and Engineering Xi'an University of Technology, No. 5 Jinhua South Road Xi'an, Shaanxi 710048, China; E-Mail: zhangjing@xaut.edu.cn; 2 School of Computer Science and Engineering, Xi'an Technological University, No. 4 Jinhua North Road Xi'an, Shaanxi 710032, China; E-Mail: mujing1977@163.com

**Keywords:** nonlinear state estimation, divided difference filter, maximum likelihood surface, target tracking

## Abstract

A new filter named the maximum likelihood-based iterated divided difference filter (MLIDDF) is developed to improve the low state estimation accuracy of nonlinear state estimation due to large initial estimation errors and nonlinearity of measurement equations. The MLIDDF algorithm is derivative-free and implemented only by calculating the functional evaluations. The MLIDDF algorithm involves the use of the iteration measurement update and the current measurement, and the iteration termination criterion based on maximum likelihood is introduced in the measurement update step, so the MLIDDF is guaranteed to produce a sequence estimate that moves up the maximum likelihood surface. In a simulation, its performance is compared against that of the unscented Kalman filter (UKF), divided difference filter (DDF), iterated unscented Kalman filter (IUKF) and iterated divided difference filter (IDDF) both using a traditional iteration strategy. Simulation results demonstrate that the accumulated mean-square root error for the MLIDDF algorithm in position is reduced by 63% compared to that of UKF and DDF algorithms, and by 7% compared to that of IUKF and IDDF algorithms. The new algorithm thus has better state estimation accuracy and a fast convergence rate.

## 1. Introduction

The problem of estimating the state of a nonlinear stochastic system from noisy measurement data has been the subject of considerable research interest during the past few years. Up to now the extended Kalman filter (EKF) has unquestionably been the dominating state estimation technique [1,2]. The EKF linearizes both the nonlinear process and the measurement dynamics with a first-order Taylor series expansion about the current state estimate. However, its accuracy depends heavily on the severity of nonlinearities. The EKF may introduce large errors and even give a divergent estimate when the nonlinearities become severe [3,4]. To improve the estimation accuracy, the second-order EKF proposed retains the Taylor series expansion up to the second term. The second–order EKF generally improves estimation accuracy, but at the expense of an increased computational burden [5]. Another attempt to improve the performance of the EKF involves the use of an iterative measurement update; the resulting algorithm is called the Iterated Extended Kalman filter (IEKF) [6]. The basic idea of IEKF is to linearize the measurement model around the updated state rather than the predicted state. This is achieved iteratively, and it involves the use of the current measurement. The IEKF has been proven to be more accurate on the condition that the state estimate is close enough to the true value, however, this is rarely the case in practice [7]. It was pointed out in [8] that the sequence of iterations generated by the IEKF and that generated by the Gauss-Newton method were identical, thus globally convergence was guaranteed. However, the Gauss-Newton method does not ensure that it goes up the likelihood surface [9,10]. Furthermore, EKF and IEKF require Jacobians, and the second-order KF requires Jacobians and Hessians. Calculation of Jacobians and Hessians is often numerically unstable and computationally intensive. In some system, the Jacobians and Hessians do not exit, which limits the applications of EKF, second-order EKF and IEKF.

Recently, there has been development in derivative-free state estimators. The finite difference has been used in the Kalman filter framework and the resulting filter is referred to as the finite difference filter (FDF) [11]. The FDF uses the first-order difference to approximate the derivative of the nonlinear function; it may introduce large state estimation errors due to a high nonlinearity, similar to the EKF. The unscented Kalman filter (UKF) proposed in [12,13] uses a minimal set of deterministically chosen sample points to capture the mean and covariance of a Gaussian density. When propagated through a nonlinear function, these points capture the true mean and covariance up to a second-order of the nonlinear function. However, the parameters used in the UKF are required to tune finely in order to prevent the propagation of non-positive definite covariance matrix for a state vector's dimension higher than three. Another Gaussian filter, named the divided difference filter (DDF) was introduced in [14] using multidimensional Stirling's interpolation formula. It is shown in [15] that the UKF and DDF algorithms are commonly referred to as sigma point filters due to the properties of deterministic sampling and weighted statistical estimation [16], but the covariance obtained in the DDF is more accurate than that in the UKF. The iterated UKF with the variable step (IUKF-VS) in [10] proposed improved the accuracy of state estimation but its runtime was large due to its computation of the sigma points. Lastly, a relatively new technique called the particle filter (PF) uses a set of randomly chosen samples with associated weights to approximate the posterior density [17] and its variants are presented in [18]. The large number of samples required often makes the use of PF computationally expensive, and the performance of PF is crucially dependent on the selection of the proposal distribution. Table 1 lists the pro and cons of the above filters.

The DDF also shows its weakness in the state estimation due to the large initial error and high nonlinearity in the application for state estimation of maneuvering target in the air-traffic control and ballistic re-entry target. Emboldened by the superiority of DDF, the basic idea of the IEKF and the iteration termination condition based on maximum likelihood, we propose a new filter named the maximum likelihood based iterated divided difference Kalman filter (MLIDDF). The performance of the state estimation for MLIDDF is greatly improved when involving the use of the iteration measurement update in the MLIDDF and the use of the current measurement. The remainder of this paper is organized as follows: in Section 2, we develop the maximum likelihood surface based iterated divided difference Kalman filter (MLIDDF). Section 3 presents the applications of the MLIDDF to state estimation for maneuvering targets in air-traffic control and ballistic target re-entry applications and discuss the simulation results. Finally, Section 4 concludes the paper and presents our outlook on future work.

## 2. Development of Likelihood Surface Based Iterated Divided Difference Filter

### 2.1. Divided Difference Filter

Consider the nonlinear function:

(1) y=f(x)

Assuming that the random variable **x** ∈ ℝ*^n_x_^* has Gaussian density with mean **x̄** and covariance **P***_x_*. The following linear transformation of **x** is introduced:

(2) $$\text{z}={\text{S}}_{x}^{-1}\text{x}$$

The transformation matrix **S***_x_* is selected as a square Cholesky factor of the covariance matrix **P***_x_* such that ${\text{P}}_{x}={\text{S}}_{x}{\text{S}}_{x}^{T}$, so the elements of **z** become mutually uncorrelated [14]. And the function **f̃** is defined by:

(3) $$\text{y}=\text{f}({\text{S}}_{x}\text{z})=\tilde{\text{f}}(\text{z})$$

The multidimensional Stirling interpolation formula of Equation (3) about **z̄** up to second-order terms is given by:

(4) $$\text{y}\approx \tilde{\text{f}}(\bar{\text{z}})+{\tilde{D}}_{\Delta z}\tilde{\text{f}}+\frac{1}{2!}{\tilde{D}}_{\Delta z}^{2}\tilde{\text{f}}$$

The divided difference operators *D̃*_Δ_*_z_***f̃**, ${\tilde{D}}_{\Delta z}^{2}\tilde{f}$ are defined as:

(5) $${\tilde{D}}_{\Delta z}\tilde{\text{f}}=\frac{1}{l}(\sum_{j=1}^{n_{x}}\Delta z_{j}\mu_{j}\delta_{j})\tilde{\text{f}}(\bar{\text{z}})$$

(6) $${\tilde{D}}_{\Delta z}^{2}\tilde{\text{f}}=\frac{1}{l^{2}}(\sum_{j=1}^{n_{x}}\Delta z_{j}^{2}\delta_{j}^{2}+\sum_{j=1}^{n_{x}}\sum_{\begin{array}{l} i=1\\ i\neq j \end{array}}^{n_{x}}\Delta z_{j}\Delta z_{i}(\mu_{j}\delta_{j})(\mu_{i}\delta_{i}))\tilde{\text{f}}(\bar{\text{z}})$$

where Δ**z** ≜ **z** - **Z̄** and Δ*z_j_* is the *j*-th element of Δ**z**. *l* denotes a selected interval length, the optimal setting of *l* is selected as $\sqrt{3}$ under the assumption that the estimation error are Gaussian and unbiased.

The partial operators *μ* and *δ* are defined as:

(7) $$\mu_{j}\text{f}(\bar{\text{z}})=\frac{1}{2}\left[\tilde{\text{f}}(\bar{\text{z}}+\frac{l}{2}{\text{e}}_{j})+\tilde{\text{f}}(\bar{\text{z}}-\frac{l}{2}{\text{e}}_{j})\right]$$

(8) $$\delta_{j}\text{f}(\bar{\text{z}})=\tilde{\text{f}}(\bar{\text{z}}+\frac{l}{2}{\text{e}}_{j})-\tilde{\text{f}}(\bar{\text{z}}-\frac{l}{2}{\text{e}}_{j})$$

where e*_j_* is the unit column vector.

We can obtain the approximate mean, covariance and cross-covariance of **y** using Equation (4):

(9) $$\bar{\text{y}}=E[\text{y}]\approx \frac{l^{2}-n_{x}}{l^{2}}\text{f}(\bar{\text{x}})+\frac{1}{2l^{2}}\sum_{j=1}^{n_{x}}\left[\text{f}(\bar{\text{x}}+l{\text{s}}_{x,j})+\text{f}(\bar{\text{x}}-l{\text{s}}_{x,j})\right]$$

(10) $$\begin{array}{ll} {\text{P}}_{yy} & =E[(\text{y}-\bar{\text{y}}){\left(\text{y}-\bar{\text{y}}\right)}^{T}]\\ & \approx \frac{1}{4l^{2}}\sum_{j=1}^{n_{x}}\left[\text{f}(\bar{\text{x}}+l{\text{s}}_{x,j})-\text{f}(\bar{\text{x}}-l{\text{s}}_{x,j})\right]\times {\left[\text{f}(\bar{\text{x}}+l{\text{s}}_{x,j})-\text{f}(\bar{\text{x}}-l{\text{s}}_{x,j})\right]}^{T}\\ & +\frac{l^{2}-1}{4l^{4}}\sum_{j=1}^{n_{x}}\left[\text{f}(\bar{\text{x}}+l{\text{s}}_{x,j})+\text{f}(\bar{\text{x}}-l{\text{s}}_{x,j})-2\text{f}(\bar{\text{x}})\right]\times {\left[\text{f}(\bar{\text{x}}+l{\text{s}}_{x,j})+\text{f}(\bar{\text{x}}-l{\text{s}}_{x,j})-2\text{f}(\bar{\text{x}})\right]}^{T} \end{array}$$

(11) $${\text{P}}_{xy}=E[(\text{x}-\bar{\text{x}}){\left(\text{y}-\bar{\text{y}}\right)}^{T}]\approx \frac{1}{2l}\sum_{j=1}^{n_{x}}{\text{s}}_{x,j}{\left(\text{f}(\bar{\text{x}}+l{\text{s}}_{x,j})-\text{f}(\bar{\text{x}}-l{\text{s}}_{x,j})\right)}^{T}$$

where **s***_x,j_* is *j*-th column of **S***_x_*.

Consider the state estimation problem of a nonlinear dynamics system with additive noise, the *n_x_*-dimensional state vector **x***_k_* of the system evolves according to the nonlinear stochastic difference equation:

(12) $${\text{x}}_{k}=\text{f}({\text{x}}_{k-1})+{\text{w}}_{k-1}$$

and the measurement equation is given as:

(13) $${\text{z}}_{k}=\text{h}({\text{x}}_{k})+{\text{v}}_{k}$$

**w***_k_*_−1_ and **v***_k_* are assumed i.i.d. and independent of current and past states, **w***_k_*_−1_ ∼ 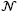
(**0, Q**_*k*−1)_, **v***_k_* ∼ 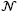
(**0, R***_k_*).

Suppose the state distribution at *k*-1 time instant is **x***_k_*_−1_∼ 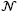
(**x̂**_*k*−1_, **P***_k_*_−1)_, and a square Cholesky factor of **P***_k_*_−1_ is **Ŝ***_x,k_*_−1_. The divided difference filter (DDF) obtained with Equations (9)–(11) can be described as follows:

Step 1. Time update
  1. Calculate matrices containing the first- and second- divided difference on the estimated state **x̂***_k_*_−1_ at *k*-1 time:
    (14) $${\text{S}}_{x\hat{x},k}^{\left(1\right)}=\{\frac{1}{2l}[\text{f}({\hat{\text{x}}}_{k-1}+l{\hat{\text{s}}}_{x,j})-\text{f}({\hat{\text{x}}}_{k-1}-l{\hat{\text{s}}}_{x,j})]\}$$
    (15) $${\text{S}}_{x\hat{x},k}^{\left(2\right)}=\{\frac{\sqrt{l^{2}-1}}{2l^{2}}[\text{f}({\hat{\text{x}}}_{k-1}+l{\hat{\text{s}}}_{x,j})+\text{f}({\hat{\text{x}}}_{k-1}-l{\hat{\text{s}}}_{x,j})-2\text{f}({\hat{\text{x}}}_{k-1})]\}$$
  2. Evaluate the predicted state and square root of corresponding covariance:
    (16) $${\bar{\text{x}}}_{k}=\frac{l^{2}-n_{x}}{l^{2}}\text{f}({\hat{\text{x}}}_{k-1})+\frac{1}{2l^{2}}\sum_{j=1}^{n_{\text{x}}}\left[\text{f}({\hat{\text{x}}}_{k-1}+l{\hat{\text{s}}}_{x,j})+\text{f}({\hat{\text{x}}}_{k-1}-l{\hat{\text{s}}}_{x,j})\right]$$
    (17) $${\bar{\text{S}}}_{x,k}=\text{Tria}([\begin{array}{ccc} {\text{S}}_{x\hat{x},k}^{\left(1\right)} & {\text{S}}_{w,k-1} & {\text{S}}_{x\hat{x},k}^{\left(2\right)} \end{array}])$$
    **ŝ**_*x,j*_ is *j*-th column of **Ŝ***_x,k_*_−1_. *Tria*() is denoted as a general triagularization algorithm and **S***_w,k_*_−1_ denotes a square-root factor of **Q***_k_*_−1_ such that ${\text{Q}}_{k-1}={\text{S}}_{w,k-1}{\text{S}}_{w,k-1}^{T}$.
Step 2. Measurement update
  1. Calculate matrices containing the first- and second-divided difference on the predicted state *x̄_k_*:
    (18) $${\text{S}}_{z\bar{x},k}^{\left(1\right)}=\{\frac{1}{2l}[\text{h}({\bar{\text{x}}}_{k}+l{\bar{\text{s}}}_{x,j})-\text{h}({\bar{\text{x}}}_{k}-l{\bar{\text{s}}}_{x,j})]\}$$
    (19) $${\text{S}}_{z\bar{x},k}^{\left(2\right)}=\{\frac{\sqrt{l^{2}-1}}{2l^{2}}[\text{h}({\bar{\text{x}}}_{k}+l{\bar{\text{s}}}_{x,j})+\text{h}({\bar{\text{x}}}_{k}-l{\bar{\text{s}}}_{x,j})-2\text{h}({\bar{\text{x}}}_{k})]\}$$
    where **s̄***_x,j_* is the *j*-th column of **S̄***_x,k_*.
  2. Evaluate the predicted measurement, square root of innovation covariance and cross-covariance:
    (20) $${\bar{\text{z}}}_{k}=\frac{l^{2}-n_{x}}{l^{2}}\text{h}({\bar{\text{x}}}_{k})+\frac{1}{2l^{2}}\sum_{j=1}^{n_{x}}\left[\text{h}({\bar{\text{x}}}_{k}+l{\bar{\text{s}}}_{x,j})+\text{h}({\bar{\text{x}}}_{k}-l{\bar{\text{s}}}_{x,j})\right]$$
    (21) $${\text{S}}_{zz,k}=\text{Tria}([\begin{array}{ccc} {\text{S}}_{z\bar{x},k}^{\left(1\right)} & {\text{S}}_{v,k} & {\text{S}}_{z\bar{x},k}^{\left(2\right)} \end{array}])$$
    (22) $${\text{P}}_{xz,k}={\bar{\text{S}}}_{x,k}{\left({\text{S}}_{z\bar{x},k}^{\left(1\right)}\right)}^{T}$$
    here **S***_v,k_* denotes a square root of **R***_k_* such that ${\text{R}}_{k}={\text{S}}_{v,k}{\text{S}}_{v,k}^{T}$.
  3. Evaluate the gain, state estimation and square root of corresponding covariance at *k* time:
    (23) $${\text{K}}_{k}=({\text{P}}_{xz,,k}/{\text{S}}_{zz,k}^{T})/{\text{S}}_{zz,k}$$
    (24) $${\hat{\text{x}}}_{k}={\bar{\text{x}}}_{k}+{\text{K}}_{k}({\text{z}}_{k}-{\bar{\text{z}}}_{k})$$
    (25) $${\hat{\text{S}}}_{x,k}=\text{Tria}([\left[\begin{array}{ccc} \left({\bar{\text{S}}}_{x,k}-{\text{K}}_{k}{\text{S}}_{z\bar{x},k}^{\left(1\right)}\right) & {\text{K}}_{k}{\text{S}}_{v,k} & {\text{K}}_{k}{\text{S}}_{z\bar{x},\;k}^{\left(2\right)} \end{array}\right]])$$
    here, the symbol “/” represents the matrix right divide operator.

### 2.2. Refining the Measurement Update Based on Divided Difference

Consider **x̄***_k_* and current measurement **z***_k_* as realization of independent random vectors with multivariate normal distributions, e.g., **x̄***_k_* ∼ 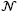
(**x**_*k*_, **P̄***_k_*) and **z***_k_* ∼ 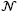
(**h**(**x***_k_*), **R***_k_*). For convenience, the two vectors are formed to a single augmented one **Z** = [**z***_k_* **x̄***_k_*]*^T^*. According to the independent assumption, we have:

(26) $$\text{Z}∼N(\text{g}({\text{x}}_{k}),\tilde{\text{Q}})$$

Here:

(27) $$\text{g}({\text{x}}_{k})={\left[\text{h}(\text{x}),{\text{x}}_{k}\right]}^{T},\tilde{\text{Q}}=\left[\begin{array}{cc} {\text{R}}_{k} & 0\\ 0 & {\bar{\text{P}}}_{k} \end{array}\right]$$

The update measurement problem becomes the one that computing the optimal state estimation and corresponding covariance given **Z, g** and **Q̃**.

Defining the objective function:

(28) $$\text{f}(\text{ξ})=\frac{1}{2}{\left\|\text{r}(\text{ξ})\right\|}^{2}$$

where **r**(*ξ*) = **S**(**Z** − **g**(*ξ*)), and **r**(*ξ*) is the second-order differentiable. Then the above update measurement becomes clearly a non-linear least squares problem.

Assuming the *i*-iterate is ${\hat{\text{x}}}_{\text{k}}^{\left(i+1\right)}$, we can obtain the following equation [8]:

(29) $${\hat{\text{x}}}_{k}^{\left(i+1\right)}={\bar{\text{x}}}_{k}+{\text{K}}_{k}^{\left(i\right)}\left({\text{z}}_{k}-\text{h}({\hat{\text{x}}}_{k}^{\left(i\right)})+{\text{H}}_{k}^{\left(i\right)}\left({\bar{\text{x}}}_{k}-{\hat{\text{x}}}_{k}^{\left(i\right)}\right)\right)$$

where ${\text{K}}_{k}^{\left(i\right)}={\bar{\text{P}}}_{k}{\text{H}}_{k}^{(i)T}{\left({\text{H}}_{k}^{(i)T}{\bar{\text{P}}}_{k}{\text{H}}_{k}^{\left(i\right)}+{\text{R}}_{k}\right)}^{-1}$.

We know the sequence of iterates generated by the IEKF and that generated by the Gauss-Newton method were identical, thus globally convergent was guaranteed. The initial state **x̄***_k_* is included in the measurement update, the value of **x̄***_k_* has a direct and large effect on the final state estimation. When the measurement model fully observes the state, the estimated state ${\hat{\text{x}}}_{\text{k}}^{\left(\text{i}\right)}$ is more approximate to the true state than the predicted state **x̄***_k_* [7]. Substituting **x̄***_k_* by ${\hat{\text{x}}}_{\text{k}}^{\left(\text{i}\right)}$ into Equation (29), hence, the following iterative formula is obtained:

(30) $${\text{x}}_{k}^{\left(i+1\right)}={\text{x}}_{k}^{\left(i\right)}+{\text{K}}_{k}^{\left(i\right)}({\text{z}}_{k}-\text{h}({\text{x}}_{k}^{\left(i\right)}))$$

Compared to Equation (29), the Equation (30) is simpler, and the two equations are identical when there is a single iteration.

Now we consider the gain:

(31) $${\text{K}}_{k}^{\left(i\right)}={\bar{\text{P}}}_{k}{\text{H}}_{k}^{(i)T}{\left({\text{H}}_{k}^{(i)T}{\bar{\text{P}}}_{k}{\text{H}}_{k}^{\left(i\right)}+{\text{R}}_{k}\right)}^{-1}$$

where the terms ${\text{H}}_{k}^{(i)T}{\bar{\text{P}}}_{k}{\text{H}}_{k}^{\left(i\right)}+{\text{R}}_{k}$ and ${\bar{\text{P}}}_{k}{\text{H}}_{k}^{(i)T}$ are approximate innovation covariance and cross-covariance obtained by linearizing the measurement equation:

(32) $${\text{P}}_{zz,k}^{\left(i\right)}={\text{H}}_{k}^{\left(i\right)}{\bar{\text{P}}}_{k}{\left({\text{H}}_{k}^{\left(i\right)}\right)}^{T}+{\text{R}}_{k}$$

(33) $${\text{P}}_{xz,k}^{\left(i\right)}={\bar{\text{P}}}_{k}{\left({\text{H}}_{k}^{\left(i\right)}\right)}^{T}$$

The terms of Equations (32) and (33) are achieved by expanding the measurement Equation (13) up to a first-order Taylor term so that the linearized error is introduced due to the high-order truncated terms. As for the highly nonlinear measurement equation, the accuracy for state estimation is decreased if the linearized error is only propagated in the Equation (30). To decrease the propagated error, we can recalculate Equations (18)–(22) to obtain the terms ${\text{P}}_{zz}^{\left(i\right)}$, ${\text{P}}_{xz}^{\left(i\right)}$ in the following way:

(34) $${\text{S}}_{zx,k}^{\left(1)(i\right)}=\{\frac{1}{2l}[\text{h}({\hat{\text{x}}}_{k}^{\left(i\right)}+l{\hat{\text{s}}}_{x,j}^{\left(i\right)})-\text{h}({\hat{\text{x}}}_{k}^{\left(i\right)}-l{\hat{\text{s}}}_{x,j}^{\left(i\right)})]\}$$

(35) $${\text{S}}_{zx,k}^{\left(2)(i\right)}=\{\frac{\sqrt{l^{2}-1}}{2l^{2}}[\text{h}({\hat{\text{x}}}_{k}^{\left(i\right)}+l{\hat{\text{s}}}_{x,j}^{\left(i\right)})+\text{h}({\hat{\text{x}}}_{k}^{\left(i\right)}-l{\hat{\text{s}}}_{x,j}^{\left(i\right)})-2\text{h}({\hat{\text{x}}}_{k}^{\left(i\right)})]\}$$

(36) $${\text{S}}_{zz,k}^{\left(i\right)}=\text{Tria}(\left[\begin{array}{ccc} {\text{S}}_{zx,k}^{\left(1)(i\right)} & {\text{S}}_{v,k} & {\text{S}}_{zx,k}^{\left(2)(i\right)} \end{array}\right])$$

(37) $${\text{P}}_{zz,k}^{\left(i\right)}={\text{S}}_{zz,k}^{\left(i\right)}{\text{S}}_{zz,k}^{(i)T}$$

(38) $${\text{P}}_{xz,k}^{\left(i\right)}={\text{S}}_{k}^{\left(i\right)}{\text{S}}_{zx,k}^{\left(1)(i\right)}$$

where ${\text{S}}_{k}^{\left(i\right)}$ is a square Cholesky factor of the covariance ${\text{P}}_{k}^{\left(i\right)}$.

Hence, we can obtain the following iterative formula:

(39) $${\hat{\text{x}}}_{k}^{\left(i+1\right)}={\text{x}}_{k}^{\left(i\right)}+{\text{P}}_{xz}^{\left(i\right)}{\left({\text{P}}_{zz}^{\left(i\right)}\right)}^{-1}\left[{\text{z}}_{k}-\text{h}({\hat{\text{x}}}_{k}^{\left(i\right)})\right]$$

### 2.3. Maximum Likelihood Based Iteration Termination Criterion

In the measurement update step of the IEKF algorithm, the inequality $\left\|{\hat{\text{x}}}_{k}^{\left(i+1\right)}-{\hat{\text{x}}}_{k}^{\left(i\right)}\right\|\leq \varepsilon$ is used as the criterion to terminate the iteration procedure, where *ε* is the predetermined threshold. The threshold *ε* is crucial to successfully using the IEKF algorithm, but selecting a proper value of *ε* is difficult [10]. The sequence of iterations generated according to the above termination condition has the property of global convergence; however, it is not guaranteed to move up the likelihood surface, so an iteration termination criterion based on maximum likelihood surface is introduced.

Consider ${\hat{\text{x}}}_{k}^{\left(i\right)}$ and **z***_k_* as the realization of independent random vectors with multivariate normal distributions, *i.e.*, ${\hat{\text{x}}}_{k}^{\left(i\right)}∼N({\text{x}}_{k},{\text{P}}_{k}^{\left(i\right)})$, **z***_k_* ∼ 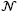
(**h**(**x***_k_*),**R***_k_*). The likelihood function of the two vectors **x***_k_* and **z***_k_* is defined as:

(40) $$\Lambda ({\text{x}}_{k},{\text{y}}_{k})=\text{const}•\exp\{-0.5\left[{\left({\text{x}}_{k}-{\hat{\text{x}}}_{k}^{\left(i\right)}\right)}^{T}{\text{P}}_{k}^{\left(-i\right)}({\text{x}}_{k}-{\hat{\text{x}}}_{k}^{\left(i\right)})+{\left({\text{z}}_{k}-\text{h}({\text{x}}_{k})\right)}^{T}{\text{R}}_{k}^{-1}({\text{z}}_{k}-\text{h}({\text{x}}_{k}))\right]\}$$

where ${\text{P}}_{\text{k}}^{\left(-i\right)}={\left({\text{P}}_{\text{k}}^{\left(i\right)}\right)}^{-1}$.

Meanwhile, the likelihood surface is defined as follows:

(41) $$J({\text{x}}_{k})={\left({\hat{\text{x}}}_{k}^{\left(i\right)}-{\text{x}}_{k}\right)}^{T}{\text{P}}_{k}^{\left(-i\right)}({\hat{\text{x}}}_{k}^{\left(i\right)}-{\text{x}}_{k})+{\left({\text{z}}_{k}-\text{h}({\text{x}}_{k})\right)}^{T}{\text{R}}_{k}^{-1}({\text{z}}_{k}-\text{h}({\text{x}}_{k}))$$

We know that the solution that maximizes the likelihood function is equivalent to minimizing the cost function *J*(**x***_k_*). The optimal value of *J*(**x***_k_*) is difficult to obtain, but the following inequality holds:

(42) $$J({\hat{\text{x}}}_{k}^{\left(i+1\right)})<J({\hat{\text{x}}}_{k}^{\left(i\right)})$$

We say that $J({\hat{\text{x}}}_{k}^{\left(i+1\right)})$ is close to the maximum likelihood surface than $J({\hat{\text{x}}}_{k}^{\left(i+1\right)})$, equivalently, ${\hat{\text{x}}}_{k}^{\left(i+1\right)}$ has a more accurate approximation than ${\hat{\text{x}}}_{k}^{\left(i\right)}$ to the minimum value of *J*(**x***_k_*) [10]. Extending Equation (42) and using ${\text{P}}_{k}^{\left(i\right)}={\text{S}}_{k}^{\left(i\right)}{\text{S}}_{k}^{(i)T}$, we immediately obtain the following inequality:

(43) $${\left({\tilde{\text{x}}}_{k}^{\left(i+1\right)}\right)}^{T}{\left({\text{S}}_{k}^{\left(i\right)}{\text{S}}_{k}^{(i)T}\right)}^{-1}{\tilde{\text{x}}}_{k}^{\left(i+1\right)}+{\left({\tilde{\text{z}}}_{k}^{\left(i+1\right)}\right)}^{T}{\text{R}}_{k}^{-1}{\tilde{\text{z}}}_{k}^{\left(i+1\right)}<{\left({\tilde{\text{z}}}_{k}^{\left(i\right)}\right)}^{T}{\text{R}}_{k}^{-1}{\tilde{\text{z}}}_{k}^{\left(i\right)}$$

where ${\tilde{\text{x}}}_{k}^{\left(i+1\right)}$ and ${\tilde{\text{z}}}_{k}^{\left(i+1\right)}$ and are defined as:

(44) $${\tilde{\text{x}}}_{k}^{\left(i+1\right)}={\hat{\text{x}}}_{k}^{\left(i+1\right)}-{\hat{\text{x}}}_{k}^{\left(i\right)}$$

(45) $${\tilde{\text{z}}}_{k}^{\left(i+1\right)}={\text{z}}_{k}-\text{h}({\hat{\text{x}}}_{k}^{\left(i+1\right)})$$

The sequence generated is guaranteed to go up the likelihood surface using Equation (43) as the criterion to iteration termination.

### 2.4. Maximum Likelihood Based Iterated Divided Difference Filter

We have now arrived at the central issue of this paper, namely, the maximum likelihood based iterated divided difference filter. Enlightened by the development of IEKF and the superiority of DDF, we can derive the maximum likelihood based iterated divided difference filter (MLIDDF) which involves the use of the iteration measurement update and the current measurement. But in view of the potential problems exhibited by the IEKF, we shall refine the covariance and cross-covariance based on divided difference and use the termination criterion which guarantees the sequence obtained moves up the maximum likelihood surface. The MLIDDF is described as follows:

Step 1. Time update
  Evaluate the predicted state **x̄***_k_* and square Cholesky factor **S̄***_k_* of the corresponding covariance **P̄***_k_* using the Equations (14)–(17).
Step 2. Measurement update
  Let ${\hat{\text{x}}}_{k}^{\left(0\right)}={\bar{\text{x}}}_{k}$ and ${\text{S}}_{k}^{\left(0\right)}={\bar{\text{S}}}_{k}$. Suppose that the *i*-th iterates are ${\hat{\text{x}}}_{k}^{\left(i\right)}$ and ${\text{S}}_{k}^{\left(i\right)}$.
  1. Evaluate the first- and second-order difference matrices using Equation (34) and (35).
  2. Evaluate the square root of innovation covariance and cross-covariance:
    (46) $${\text{S}}_{zz,k}^{\left(i\right)}=\text{Tria}(\left[\begin{array}{ccc} {\text{S}}_{zx,k}^{\left(1)(i\right)} & {\text{S}}_{v,k} & {\text{S}}_{zx,k}^{\left(2)(i\right)} \end{array}\right])$$
    (47) $${\text{P}}_{xz,k}^{\left(i\right)}={\text{S}}_{k}^{\left(i\right)}{\text{S}}_{zx,k}^{\left(1)(i\right)}$$
  3. Evaluate the gain:
    (48) $${\text{K}}_{k}^{\left(i\right)}={\text{P}}_{xz}^{\left(i\right)}/{\text{S}}_{zz,k}^{(i)T}/{\text{S}}_{zz,k}^{\left(i\right)}$$
  4. Evaluate the state and the square root of corresponding covariance:
    (49) $${\hat{\text{x}}}_{k}^{\left(i+1\right)}={\hat{\text{x}}}_{k}^{\left(i\right)}+{\text{K}}_{k}^{\left(i\right)}[{\text{z}}_{k}-\text{h}({\hat{\text{x}}}_{k}^{\left(i\right)})]$$
    (50) $${\text{S}}_{k}^{\left(i+1\right)}=\text{Tria}(\left[\begin{array}{ccc} \left({\bar{\text{S}}}_{x,k}-{\text{K}}_{k}^{\left(i\right)}{\text{S}}_{zx,k}^{\left(1)(i\right)}\right) & {\text{K}}_{k}^{\left(i\right)}{\text{S}}_{v} & {\text{K}}_{k}^{\left(i\right)}{\text{S}}_{zx,k}^{\left(2)(i\right)} \end{array}\right])$$
    where ${\hat{\text{s}}}_{x,j}^{\left(i\right)}$ is the *j*-th column of ${\text{S}}_{k}^{\left(i\right)}$.
Step 3. If the following inequality holds:
  (51) $${\left({\tilde{\text{x}}}_{k}^{\left(i+1\right)}\right)}^{T}{\left({\text{S}}_{k}^{\left(i\right)}{\text{S}}_{k}^{(i)T}\right)}^{-1}{\tilde{\text{x}}}_{k}^{\left(i+1\right)}+{\left({\tilde{\text{z}}}_{k}^{\left(i+1\right)}\right)}^{T}{\text{R}}_{k}^{-1}{\tilde{\text{z}}}_{k}^{\left(i+1\right)}<{\left({\tilde{\text{z}}}_{k}^{\left(i\right)}\right)}^{T}{\text{R}}_{k}^{-1}{\tilde{\text{z}}}_{k}^{\left(i\right)}$$
  The iteration returns to Step 2; otherwise continue to Step 4. ${\tilde{\text{x}}}_{k}^{\left(i+1\right)}$, ${\tilde{\text{z}}}_{k}^{\left(i+1\right)}$ are defined in the Equations (44) and (45).
Step 4. If the inequality is not satisfied or if *i* is too large (*i* > *N*_max_), and the ultimate state estimation and square root of corresponding covariance at *k* time instant are:
  (52) $${\hat{\text{x}}}_{k}={\hat{\text{x}}}_{k}^{\left(N_{\max}\right)}$$
  (53) $${\text{S}}_{k}={\text{S}}_{k}^{\left(N_{\max}\right)}$$

The MLIDDF algorithm has the virtues of free-derivative and better numerical stability. The measurement update of MLIDDF algorithm is transformed to a nonlinear least-square problem; the optimum state estimation and covariance are solved using Gauss-Newton method, so the MLIDDF algorithm has the same global convergence as the Gauss-Newton method. Moreover, the iteration termination condition that makes the sequence move up the maximum likelihood surface is used in the measurement update process.

## 3. Simulation and Analysis

In this section, we reported the experimental results obtained by applying the MLIDDF to the nonlinear state estimation of a maneuvering target in an air-traffic control scenario and a ballistic target re-entry scenario. To demonstrate the performance of the MLIDDF algorithm we compared its performance against the UKF, DDF, and the iterated UKF (IUKF) and iterated DDF (IDDF), both using a traditional iteration strategy.

### 3.1. Maneuvering Target Tracking in the Air-Traffic Control Scenario

We consider a typical air-traffic control scenario, where an aircraft executes a maneuvering turn in a horizontal plane at a constant, but unknown turn rate Ω. The kinematics of the turning motion can be modeled by the following nonlinear process equation [2,19]:

(54) $${\text{x}}_{k}=\left(\begin{array}{ccccc} 1 & \frac{\sin\Omega T}{\Omega } & 0 & -\frac{1-\cos\Omega T}{\Omega } & 0\\ 0 & \cos\Omega T & 0 & -\sin\Omega T & 0\\ 0 & \frac{1-\cos\Omega T}{\Omega } & 1 & \frac{\sin\Omega T}{\Omega } & 0\\ 0 & \sin\Omega T & 0 & \cos\Omega T & 0\\ 0 & 0 & 0 & 0 & 1 \end{array}\right){\text{x}}_{k-1}+{\text{w}}_{k-1}$$

where the state of the aircraft **x** = [*x ẋ y ẏ* Ω]; *x* and *y* denote positions, and *ẋ* and *ẏ* denote velocities in the *x* and *y* directions, respectively; *T* is the time-interval between two consecutive measurements;

The process nose **w***_k_*_−1_∼ 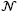
(**0, Q**) with a nonsingular covariance:

(55) $$\text{Q}=\left(\begin{array}{ccc} q_{1}\text{π} & 0 & 0\\ 0 & q_{1}\text{π} & 0\\ 0 & 0 & q_{2}T \end{array}\right),\text{π}=\left(\begin{array}{cc} T^{3}/3 & T^{2}/2\\ T^{2}/2 & T \end{array}\right)$$

The parameters *q*_1_ and *q*_2_ are related to process noise intensities. A radar is fixed at the origin of the place and equipped to measure the range, *r* and bearing, *θ*. Hence, the measurement equation is written:

(56) $$\left(\begin{array}{c} r_{k}\\ \theta_{k} \end{array}\right)=\left(\begin{array}{c} \sqrt{x_{k}^{2}+y_{k}^{2}}\\ \tan^{-1}(y_{k}/x_{k}) \end{array}\right)+{\text{v}}_{k}$$

where the measurement noise **v***_k_* ∼ 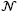
(**0, R**) and $\text{R}=\text{diag}([\sigma_{r}^{2}\sigma_{\theta }^{2}])$.

The parameters used in this simulation were the same as those in [19]. To tracking the maneuvering aircraft we use the proposed MLIDDF algorithm and compare its performance against the DDF. We use the root-mean square error (*RMSE*) of the position, velocity and turn rate to compare the performances of two nonlinear filters. For a fair comparison, we make 250 independent Monte Carlo runs. The RMSE in position at time *k* is defined as:

(57) $${\text{RMSE}}_{p}(k)=\sqrt{\frac{1}{N}\sum_{i=1}^{N}{\left(x_{k}^{\left(i\right)}-{\hat{x}}_{k}^{\left(i\right)}\right)}^{2}+{\left(y_{k}^{\left(i\right)}-{\hat{y}}_{k}^{\left(i\right)}\right)}^{2}}$$

where $\left(x_{k}^{\left(i\right)},y_{k}^{\left(i\right)}\right)$ and $\left({\hat{x}}_{k}^{\left(i\right)},{\hat{y}}_{k}^{\left(i\right)}\right)$ are the true and estimated positions at the *i*-th Monte Carlo run. Similarly to RMSE in position, we may also write formulas of *RMSE* in velocity and turn rate.

**Figure 1.**
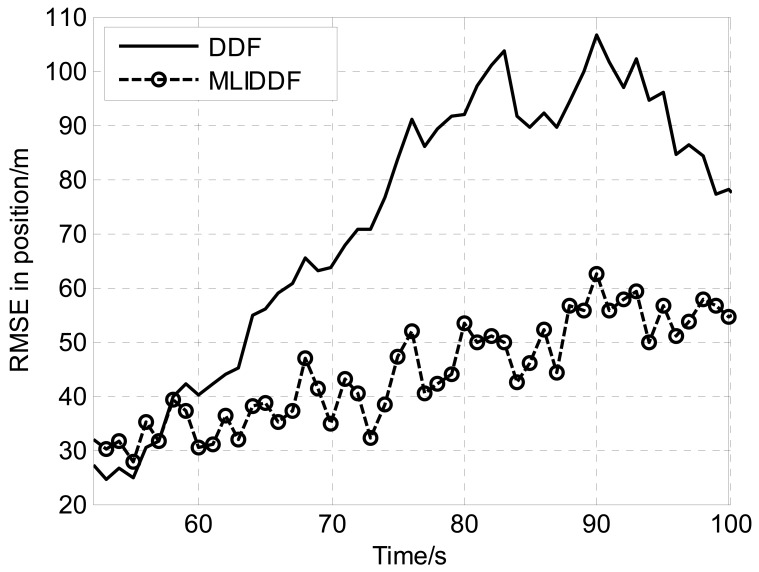
RMSE in position for DDF and MLIDDF.

**Figure 2.**
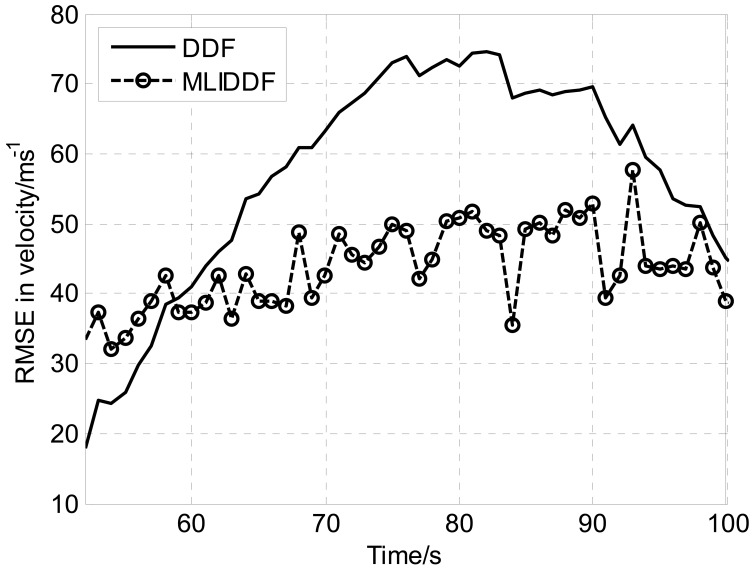
RMSE in velocity for DDF and MLIDDF.

**Figure 3.**
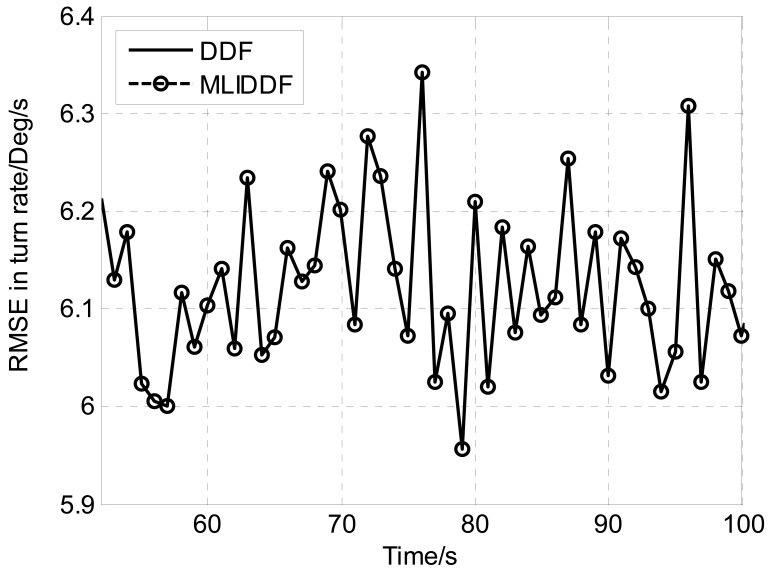
RMSE in turn rate for DDF and MLIDDF.

Owing to the fact that the filters is sensitive to initial state estimation, Figures 1–3 show the *RMSE*s in position, velocity and turn rate, respectively for DDF and MLIDDF in an interval of 50–100 s. As can be seen in Figures 1–3, the MLIDDF significantly outperforms the DDF.

**Figure 4.**
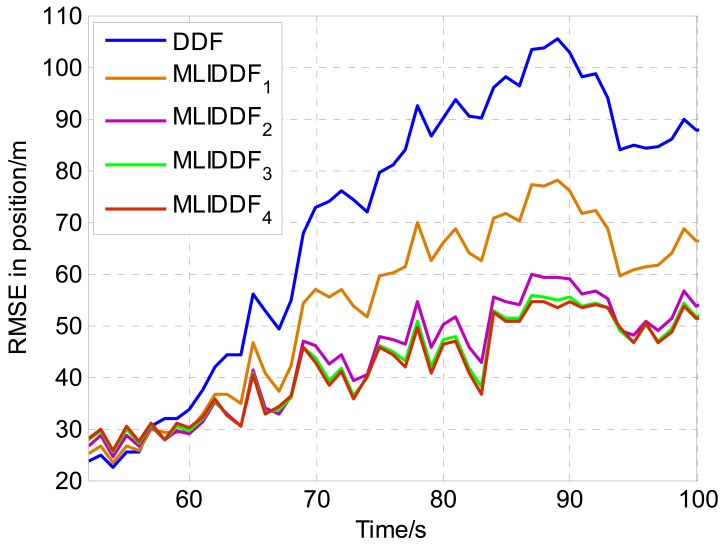
RMSE in position for MLIDDFs with various iterate numbers (MLIDDF_1_ with iterate numbers 2, MLIDDF_2_ with 5, MLIDDF_3_ with 8, MLIDDF_4_ with 10).

In order to analyze the impact of iteration numbers on performance of the MLIDDF algorithm, the MLIDDFs with various iteration numbers are applied to position estimation of maneuvering target. Figure 4 shows the *RMSE*s of DDF and MLIDDFs with various numbers in position. From Figure 4, the *RMSE* in position for the MLIDDF with iteration number 2 begins to decrease largely comparable to that of DDF. The *RMSE* in position for MLIDDF with iteration number 5 significantly reduce, and the MLIDDF algorithm has very fast convergence rate. The *RMSE* of the position for MLIDDF decreases slowly when the iteration number is greater than 8; the reason is that the sequence generated has basically reached the maximum likelihood surface when the iteration termination condition is met.

### 3.2. State Estimation of Reentry Ballistic Target

#### 3.2.1. Simulation Scene

**Figure 5.**
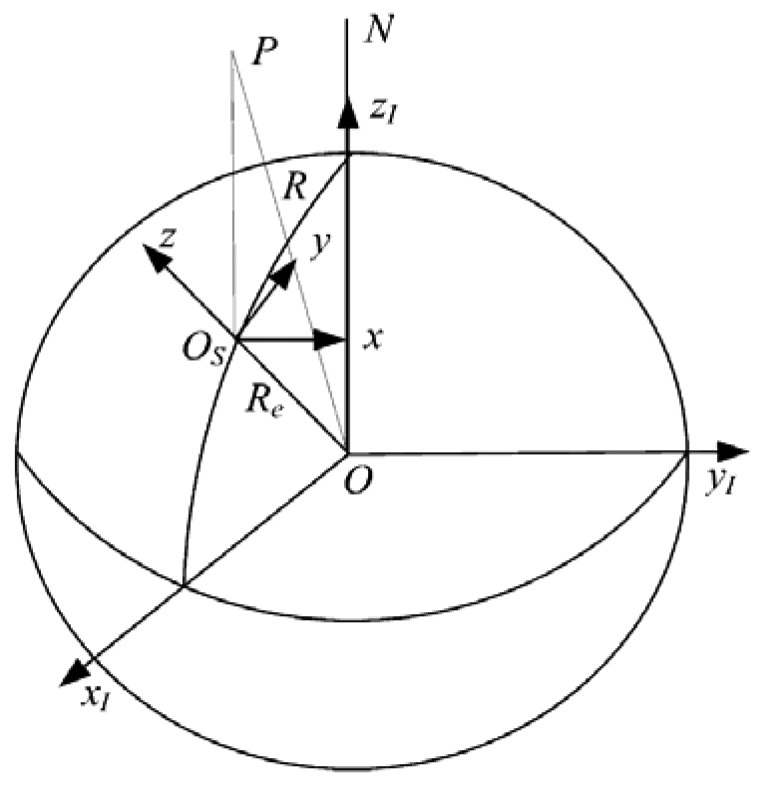
Geometry of radar and BTR.

The relative location of the ballistic target re-entry (BTR) and the radar are shown in Figure 5. The inertial coordinate system (ECI-CS) *Ox_I_y_I_z_I_* shown in Figure 5 is a right-handed system with the origin *O* at the Earth's center, axis *Ox_I_* pointing in the vernal equinox direction, axis *Oz_I_* pointing in the direction of the North Pole *N*. Its fundamental plane *Ox_I_y_I_* coincides with the Earth's equatorial plane.

Assume that the radar is situated at the surface of the Earth and considering the orthogonal coordinate reference system named East-North-Up coordinates system (ENU-CS) *O_s_xyz* has its origin at the location of the radar. In this system, *z* is directed along the local vertical and *x* and *y* lie in a local horizontal plane, with *x* pointing east and *y* pointing north. Assuming that the Earth is spherical and non-rotating and the forces acting on the target are only gravity and drag [20], we can derive the following state equation according to the kinematics of the ballistic re-entry object in the reentry phase in ENU-CS:

(58) $${\text{x}}_{k}=\text{Φ}{\text{x}}_{k-1}+\text{G}\psi ({\text{x}}_{k-1})+{\text{w}}_{k-1}$$

where **x***_k_* = [*x_k_ ẋ_k_ y_k_ ẏ_k_ z_k_ ż_k_ β_k_*]*T* is the state of ballistic target re-entry, and:

(59) $$\text{Φ}=\left[\begin{array}{cccc} \phi & 0 & 0 & 0\\ 0 & \phi & 0 & 0\\ 0 & 0 & \phi & 0\\ 0 & 0 & 0 & 1 \end{array}\right],\phi =\left[\begin{array}{cc} 1 & T\\ 0 & 1 \end{array}\right]$$

(60) $$\text{G}=\left[\begin{array}{ccc} \tau & 0 & 0\\ 0 & \tau & 0\\ 0 & 0 & \tau \\ 0 & 0 & 0 \end{array}\right],\tau =\left[\begin{array}{c} T^{2}/2\\ T \end{array}\right]$$

(61) $$\psi ({\text{x}}_{k-1})=\left[\begin{array}{c} -\frac{\rho (h_{k-1})}{2\beta_{k-1}}V_{k-1}{\dot{x}}_{k-1}-\frac{\mu x_{k-1}}{r_{k-1}^{3}}\\ -\frac{\rho (h_{k-1})}{2\beta_{k-1}}V_{k-1}{\dot{y}}_{k-1}-\frac{\mu y_{k-1}}{r_{k-1}^{3}}\\ -\frac{\rho (h_{k-1})}{2\beta_{k-1}}V_{k-1}{\dot{z}}_{k-1}-\frac{\mu (z_{k-1}+R_{e})}{r_{k-1}^{3}} \end{array}\right]$$

where

(62) $$\left\{ \begin{array}{l} r_{k-1}=\sqrt{x_{k-1}^{2}+y_{k-1}^{2}+{\left(z_{k-1}+R_{e}\right)}^{2}}\\ V_{k-1}=\sqrt{{\dot{x}}_{k-1}^{2}+{\dot{y}}_{k-1}^{2}+{\dot{z}}_{k-1}^{2}}\\ h_{k-1}=r_{k-1}-R_{e} \end{array}\right.$$

*T* is the time interval between radar measurements, *β* is the ballistic coefficient (kg/m^2^), *μ* and *R*_e_ are the Earth's gravitational constant and average Earth radius, respectively. Below 90 km at height, the air density *ρ(h)* is approximately modeled as an exponentially decaying function of height, *ρ*(*h*) = *c*_1_*e*^−*c*_2_*h*^ (*c*_1_, *c*_2_ are constant, specifically, *c*_1_ = 1.227, *c*_2_ = 1.093 × 10^−4^ for h < 9,144 m, and *c*_1_ = 1.754, *c*_2_ = 1.49 × 10^−4^ for *h* ≥ 9,144 m) [21].

Process noise **w***_k_* is assumed to be white noise with zero mean; its covariance is approximately modeled as [2]:

(63) $${\text{Q}}_{k}=\left[\begin{array}{cccc} q_{1}{\text{θ}}_{1} & 0 & 0 & 0\\ 0 & q_{1}{\text{θ}}_{1} & 0 & 0\\ 0 & 0 & q_{1}{\text{θ}}_{1} & 0\\ 0 & 0 & 0 & q_{2}T \end{array}\right],\text{θ}=\left[\begin{array}{cc} \overset{T^{3}}{\;}/\underset{3}{\;} & \overset{T^{2}}{\;}/\underset{2}{\;}\\ \overset{T^{2}}{\;}/\underset{2}{\;} & T \end{array}\right]$$

where *q*_1_ (in m^2^/s^3^) and *q*_2_ (in kg^2^/m^4^s) are the intensity of noise.

According to relative geometry, the measurement equation in the ENU-CS is described as:

(64) $${\text{z}}_{k}=\text{h}({\text{x}}_{k})+{\text{v}}_{k}$$

here **z***_k_* = [*R_k_ E_k_ A_k_*]*^T^*, and:

(65) $$R_{k}=\sqrt{x_{k}^{2}+y_{k}^{2}+z_{k}^{2}}+v_{R}$$

(66) $$E_{k}=tan^{-1}z_{k}/\sqrt{x_{k}^{2}+y_{k}^{2}}+v_{E}$$

(67) $$A_{k}=tan^{-1}y_{k}/x_{k}+v_{A}$$

The measurement noise **v***_k_* is assumed to be white noise with zero mean and covariance:

(68) $${\text{N}}_{k}=\text{diag}(\left[\begin{array}{ccc} \sigma_{R}^{2} & \sigma_{E}^{2} & \sigma_{A}^{2} \end{array}\right])$$

where *σ_R_, σ_E_, σ_A_*, are the error standard deviations of range, elevation and azimuth, respectively. It is independent of the process noise **w***_k_* and initial state **x**_0_.

#### 3.2.2. Numerical Results and Analysis

The parameters used in simulation were: *T* = 0.1 s, *q*_1_ = 5 m^2^/s^3^, *q*_2_ = 5 kg^2^/m^4^s. The initial position and magnitude of the velocity: *x*_0_ = 232 km, *y*_0_ = 232 km, *z*_0_ = 90 km and *v*_0_ = 3,000 m/s, and the initial elevation and azimuth angle: *E*_0_ = 7π/6 and *A*_0_ = π/4. The ballistic coefficient was selected as *β* = 4,000 kg/m^2^. The error standard deviations of the measurements were selected as *σ_R_* = 100 m, *σ_E_* = 0.017 rad, and *σ_A_* = 0.017 rad. A threshold *ε* = 10 was set in the IUKF and IDDF, and a maximum iteration number *N*_max_ = 8 was predetermined.

From the above parameters given, we can obtain the initial true state:

$$x_{0}=[232km\;-1837m/s\;232km\;-1837m/s\;90km\;-1500m/s\;4000kg/m^{2}]$$

and we select the corresponding covariance as: **P**_0_ = *diag*[100^2^ 50^2^ 100^2^ 50^2^ 100^2^ 50^2^ 200^2^].

To compare the performance of the various filter algorithms, we also use *RMSE* in the position, velocity and ballistic coefficient. The position *RMSE* at *k* time of the ballistic target re-entry is defined as:

(69) $${\text{RMSE}}_{p}(k)=\sqrt{\frac{1}{N}\sum_{i=1}^{N}\left({\left(x_{k}^{\left(i\right)}-{\hat{x}}_{k}^{\left(i\right)}\right)}^{2}+{\left(y_{k}^{\left(i\right)}-{\hat{y}}_{k}^{\left(i\right)}\right)}^{2}+{\left(z_{k}^{\left(i\right)}-{\hat{z}}_{k}^{\left(i\right)}\right)}^{2}\right)}$$

where $\left(x_{k}^{\left(i\right)},y_{k}^{\left(i\right)},z_{k}^{\left(i\right)}\right)$ and $\left({\hat{x}}_{k}^{\left(i\right)},{\hat{y}}_{k}^{\left(i\right)},{\hat{z}}_{k}^{\left(i\right)}\right)$ are the true and estimated position at the *i*-th Monte Carlo run, *N* is the Monte Carlo runs. Similarity to the *RMSE* in position we may also write formulas of the *RMSE* in velocity and ballistic coefficient.

**Figure 6.**
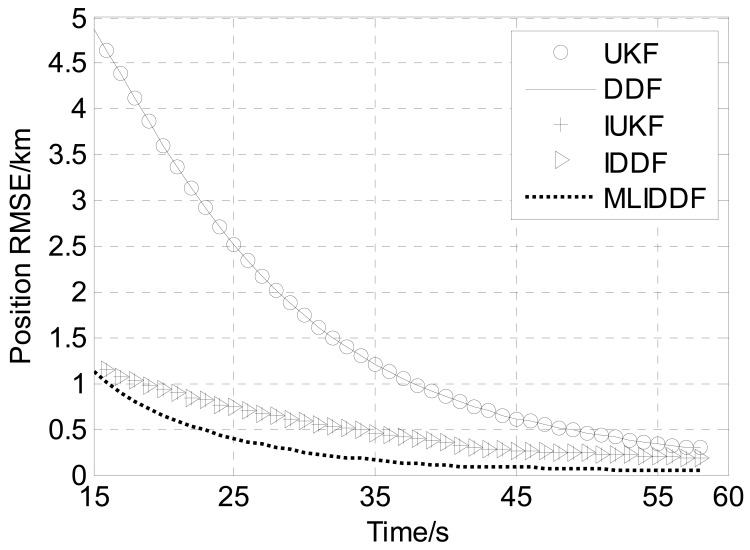
Position *RMSE*s for various filters.

**Figure 7.**
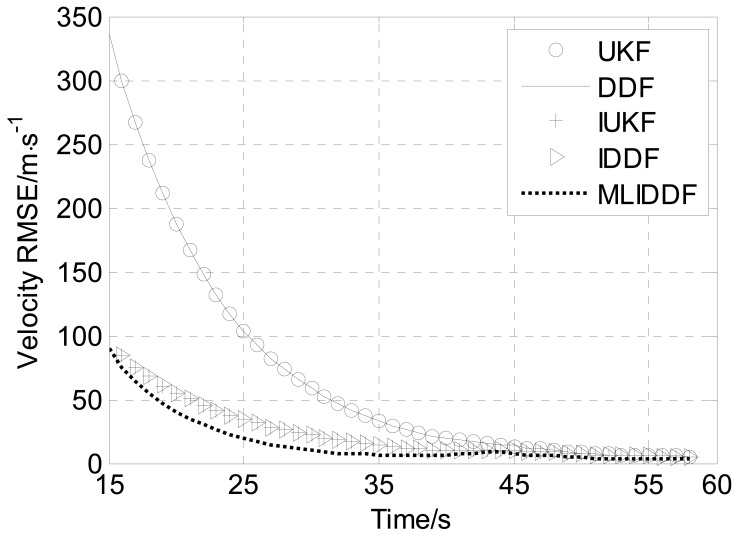
Velocity *RMSE*s for various filters.

**Figure 8.**
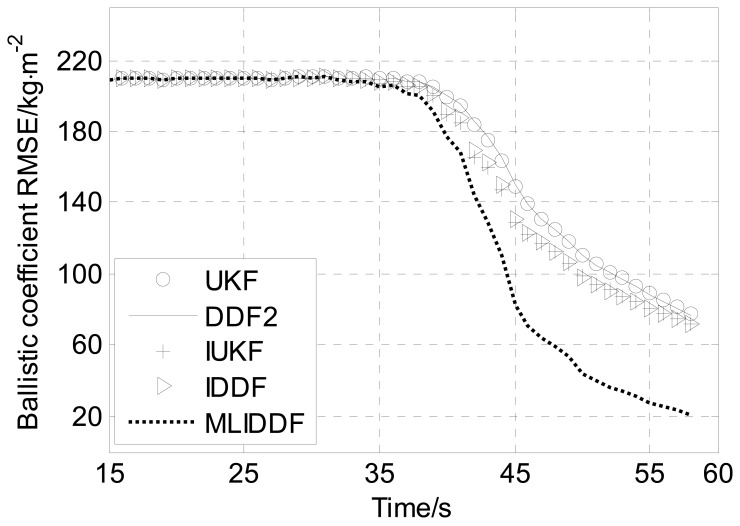
Ballistic coefficient *RMSE*s for various filters.

Figures 6–8 shows the position, velocity and ballistic coefficient *RMSE*s, respectively, for the various filters in an interval of 15–58 s. The initial state estimate **x̂**_0_ is chosen randomly from **x̂**_0_ ∼ 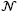
(**x**_0_, **P**_0_) in each run. All the filters are initialized with the same condition in each run. We make 100 independent Monte Carlo runs.

From Figure 6 we can see that the position *RMSE* of the MLIDDF is much less than the UKF and DDF because of the use of the current measurement in the step of the iteration measurement update of the MLIDDF, and is less than the IUKF and IDDF owing to involving the proposed iteration strategy, so the estimates provided by the MLIDDF are markedly better than those of the UKF and DDF algorithms, and are better than those of IUKF and IDDF algorithms. The MLIDDF also shows a significant improvement over the other filters in the estimation of the velocity, as evidenced by Figure 7.

As to the estimation of the ballistic coefficient, in the Figure 8, we can see that there is no improvement in the *RMSE* in the initial interval of the observation period (t < 35 s) because there is no effective information about it, while in the remaining period (35 s < t < 58 s) the ballistic coefficient *RMSE* is decreased because the effective information about the ballistic coefficient from the latest measurement is fully used. Especially, Figure 8 illustrates that toward the end of the trajectory the estimates provided by the MLIDDF are markedly better than those of the UKF, DDF, IUKF and IDDF algorithms.

Meanwhile, we observe from Figures 6–8 that the UKF and DDF have almost the same performance in the problem and the performance of IUKF and IDDF algorithms are almost identical.

For further comparison of performance for the various filters, the average accumulated position mean-square root error (*AMSRE*) is defined as follows:

(70) $${\text{AMSRE}}_{p}=\frac{1}{N}\sum_{i=1}^{N}\sqrt{\left[\frac{1}{M}\sum_{k=1}^{M}\left[{\left(x_{k}^{\left(i\right)}-{\hat{x}}_{k}^{\left(i\right)}\right)}^{2}+{\left(y_{k}^{\left(i\right)}-{\hat{y}}_{k}^{\left(i\right)}\right)}^{2}+{\left(z_{k}^{\left(i\right)}-{\hat{z}}_{k}^{\left(i\right)}\right)}^{2}\right]\right]}$$

where *M* is the total number of measurement data points. The formulas of the velocity and ballistic coefficient *AMSREs* can be written similar to the *AMSRE_p_*. The position, velocity and ballistic coefficient *AMSREs* for the various filters are listed in Table 2. Table 3 lists the runtimes of the various filters.

From Table 2, it is seen that the position *AMSRE* for the MLIDDF algorithm is reduced by 62% compared to the UKF and DDF, by 7% compared to IUKF and IDDF. The velocity *AMSRE* for the MLIDDF algorithm is reduced by 23% compared to the UKF and DDF algorithms. The ballistic coefficient *AMSRE* for the MLIDDF algorithm is reduced by 7% compared to the UKF and DDF algorithms. Although the ballistic coefficient *AMSRE* for the MLIDDF algorithm has no significant reduction, we can see that it is reduced significantly compared to the UKF, DDF, IUKF and IDDF algorithms, hence, the MLIDDF is preferred over the other filters in the light of position, velocity and ballistic coefficient *AMSRE*s.

From Table 3, we can see the runtime of MLIDDF is less than that of the IUKF algorithm, and is more than those of UKF, DDF, and IDDF algorithms, so the accuracy of the MLIDDF algorithm is improved at the cost of an increased computational burden. Meanwhile, we can observe the *AMSREs* for the UKF and DDF in position, velocity and ballistic coefficient are almost identical, and the *AMSREs* of the IUKF and IDDF are almost the same. Therefore, on the basis of the simulation results presented in Figures 6–8 and Table 2, one can draw the conclusion that the MLIDDF yields a superior performance over the other filters.

## 4. Conclusions and Future Work

In this study, we provide the maximum likelihood based iterated divided difference filter which inherits the virtues of the divided difference filter and contains the iteration process in the measurement update step. The sequence obtained is guaranteed to move up the likelihood surface using the iteration termination condition based on the maximum likelihood surface. The maximum likelihood based iterated divided difference is implemented easily and is derivative-free. We apply the new filter to state estimation for a ballistic target re-entry scenario and compare its performance against the unscented Kalman filter, divided difference filter, iterated unscented Kalman filter and iterated divided difference filter with the traditional termination criteria. Simulation results demonstrate that the maximum likelihood-based iterated divided difference is much more effective than the other filters. The maximum likelihood-based iterated divided difference greatly improves the performance of state estimation and has a shorter convergence time.

Future work may focus on the applications of the maximum likelihood iteration divided difference filter to remove the outliers which is a serious deviation from the sample and caused by blink and subjective eye movement in video nystagmus signal samples of pilot candidates.

## Figures and Tables

**Table 1.** Pro and cons for various filters.

**Various filters**	**Advantage**	**Disadvantage**
EKF	Less runtime	Calculation of Jacobian
Second-order EKF	high accuracy	Calculation of Jacobian and Hessian
IEKF	high accuracy	Calculation of Jacobian
UKF	Derivative-free	more runtime
IUKF-VS	Derivative-free, high accuracy	more runtime
FDF	Derivative-free, Less runtime	Lower accuracy
DDF	Derivative-free	Low accuracy
PF	Derivative-free	Heavy computational burden

**Table 2.** *AMSRE*s for various filters.

**Various algorithms**	***AMSRE_p_(m)***	***AMSRE_v_(m/s)***	***AMSRE_β_(kg/m^2^)***
UKF	2521.684	329.911	155.735
DDF	2521.573	329.903	155.276
IUKF	1035.340	259.173	149.603
IDDF	1035.273	260.771	149.756
MLIDDF	968.746	255.916	144.953

**Table 3.** Runtimes of various filters.

**Various algorithms**	**Runtime(*s*)**
UKF	1.0840
DDF	0.2888
IUKF	1.9918
IDDF	0.5133
MLIDDF	1.3074
